# Supplementary material for: How the visual brain can learn to parse images using a multiscale, incremental grouping process
Source: PLoS Comput Biol. 2026 Apr 15;22(4):e1014193. doi: 10.1371/journal.pcbi.1014193 (PMC13095124; doi:10.1371/journal.pcbi.1014193)
Supplement: S1 Text — (DOCX) [file pcbi.1014193.s001.docx]

**Alternatives for the disinhibitory connection scheme**

To examine the role of disinhibition in stable long-range grouping, we compared the present architecture to previous networks [17] in which excitatory units directly activated neighboring excitatory units, without an intervening inhibitory circuit. When implemented with ReLU nonlinearities, the excitatory recurrent networks were unstable because activity is unbounded. Replacing the ReLU with a squashing nonlinearity prevented runaway excitation but introduced a different limitation because the magnitude of the response enhancement decreased progressively along the target curve. These networks failed to reliably trace curves longer than 12 pixels, consistent with previous findings [17].

In contrast, the disinhibitory circuit enabled stable propagation of enhanced activity without attenuation, allowing the network to generalize to curves substantially longer than those presented during training. The panel A of the supplemental figure compares the generalization performance of the model developed in ref. [17], which combined excitatory connectivity with a squashing nonlinearity, to the present disinhibitory model. When trained to trace curves up to length *N*, the performance of the excitatory model deteriorated for curves longer than *N* + 4. By contrast, the disinhibitory model maintains high performance as curve length increases.

This difference becomes apparent when comparing activity between these network types (Supplemental Figure, panels B,C). In the disinhibitory model, pyramidal units are either suppressed by SOM units of fully disinhibited once the propagating signal reaches them. This bistable regime prevents attenuation of the activity difference between target and distractor curves so that the contrast between grouped and non-grouped elements remains stable over distance. In contrast, in models with excitatory units with a squashing nonlinearity, the activity difference decreases along the target curve and eventually vanishes, preventing reliable discrimination from the target curve.


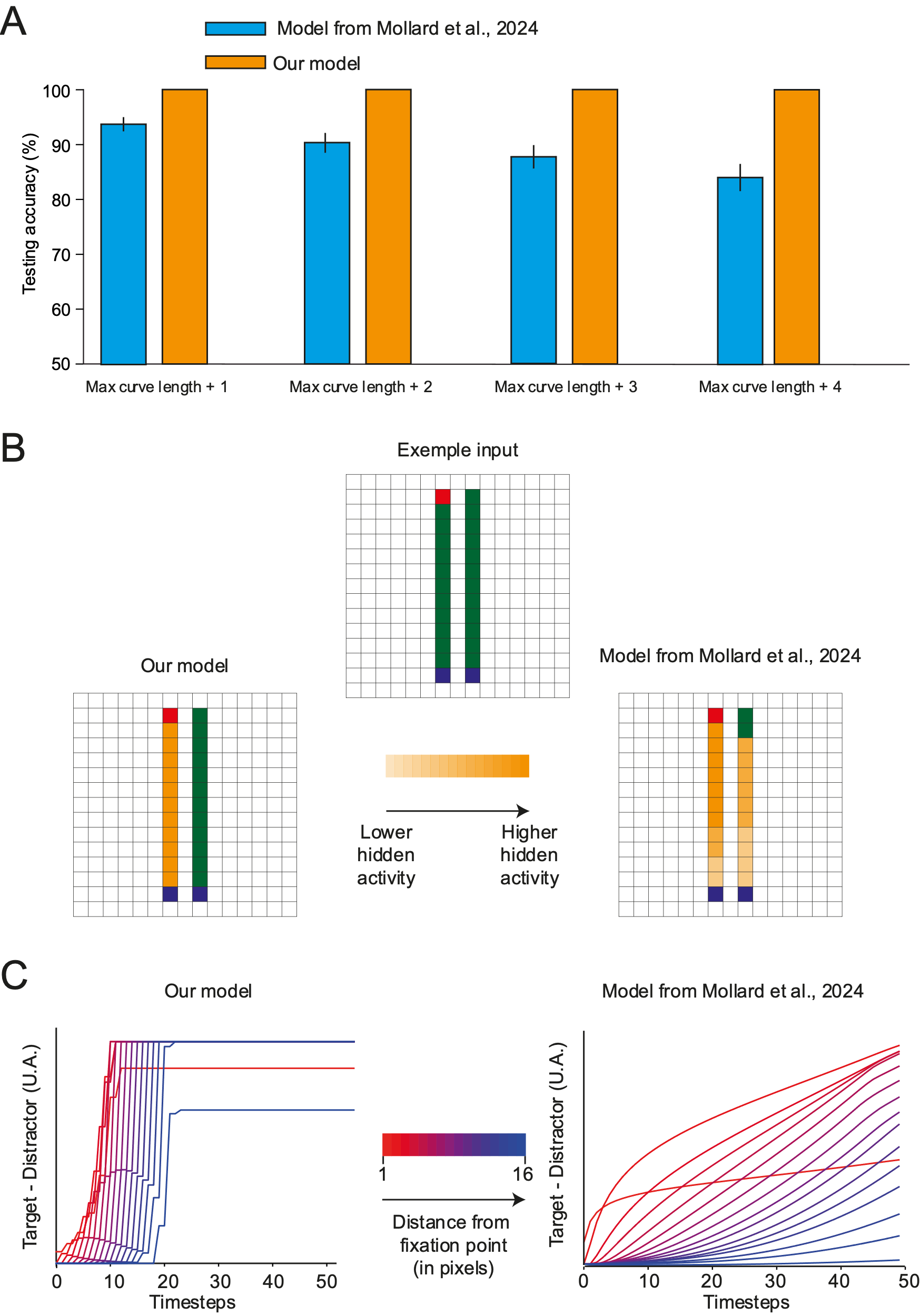


**Figure A.** Comparison of the model with disinhibition to models composed of excitatory units and a squashing non-linearity. **A.** Generalization to curves longer than those presented during training. Networks were trained up to a maximum curve length *N* and tested on curves of length *N* + 1 to *N* + 4. The previous model (ref. [17]; blue) shows a progressive decline in performance as curve length increases. In contrast, the present disinhibitory model (orange) maintains near-perfect accuracy across all tested lengths, demonstrating superior generalization. Error bars indicate standard error across networks. **B.** Top, example stimulus. Bottom, activity in the disinhibitory network (left) and the previous model (ref. [17]; right). In the disinhibitory model, enhanced activity exclusively propagates along the target curve (connected to the red pixel) without attenuation, while units responding to the distractor curve remain fully suppressed. In contrast, in models with the excitatory connection scheme with a squashing nonlinearity, activity spreads along both curves, progressively diminishing the contrast between the target and distractor curves. **C.** Difference in activity between units with RFs on the target and distractor curves, as function of time. Curves are color-coded by distance from the fixation point (red = near, blue = far). **left,** In the disinhibitory network, the activity difference reaches a stable plateau at all distances. The lower plateau observed for the first and last pixels arises because each is flanked by only one active neighbor, resulting in reduced recurrent input to counteract inhibition, rather than reflecting a true decrease in activity difference. **right,** In the purely excitatory model, the activity difference decreases with curve length, eventually approaching zero for distant units, explaining the failure to generalize to longer curves.
