# Supplementary material for: How the visual brain can learn to parse images using a multiscale, incremental grouping process
Source: PLoS Comput Biol. 2026 Apr 15;22(4):e1014193. doi: 10.1371/journal.pcbi.1014193 (PMC13095124; doi:10.1371/journal.pcbi.1014193)
Supplement: Attachment — Submitted filename: response_to_reviewers.docx [file pcbi.1014193.s002.docx]

**Reviewer 1**

**General comment**

R1-0) The authors present an artificial neural network (ANN) implementation of the growth-cone model in order to explain neurophysiological and psychophysical findings. They trained an ANN comprising two separate units and a disinhibitory circuit within the recurrent network, modelling the differences in activity propagation and reaction times for curve-tracing and object-parsing tasks. The advantage of the proposed network is that it is biologically plausible and stable, and in the case of curve tracing, it can be extended to cover long curves. The paper is clear, and the results demonstrate that this architecture can explain previous findings using a biologically plausible neural network. Based on my limited knowledge of incremental grouping, this work appears novel and worthy of acceptance for publication in PLOS Computational Biology.

R1-0 reply) We thank the reviewer for this evaluation and encouraging assessment.

Minor Comments :

R1-1) Figure 3D: the inter model variability is never mentioned, it can be interesting to plot error bars and comment a bit more on whether all models had the same strategy and results (if it was studied)

R1-1 reply) All models learned a similar strategy. We suspect that the deterministic nature of the task and the constraints imposed on the model resulted in the absence of substantial variability in processing times across the five models. Hence, we could not plot meaningful error bars in Fig. 3D. We have now clarified this point in the manuscript (p. 19, lines 427-428).

R1-2) Figure 4C: authors observe the explained variance evolution when decreasing the number of scales. Is it complicated to increase the number of scales ? (I guess removing some layers of the trained network is easy compare to retraining a bigger model but increasing the amounts of scale and observing an increase in explained variance would provide a stronger proof than an ablation study)

R1-2 reply) We trained networks with 2, 3 and 4 layers and observed that more scales increase the explained variance. Adding a fifth scale would produce a layer with very large receptive fields (across scales the receptive fields increase in size by a factor of three in the two spatial dimensions), making it unlikely that the network would be able explain more variance.

R1-3) I wonder to what extend the training procedure impacts the model's performance and if a hard-wired architecture for both the FF model (that detects either connected and colinear units or all active units) and the RNN propagating activity only to the neighbors with local connections can also easily account for the results. This question is a bit redundant with the question on how variable models are. Whether if the heuristics used here constraint the model in a way that learning may not be necessary.

# R1-3 reply) Models with hard-wired architectures have previously been shown to learn similar tasks in a multiscale manner (see, for example, ‘A multi-scale neurodynamic implementation of incremental grouping’, Domijan et al. 2022 and ‘A model of thalamo-cortical interaction for incremental binding in mental contour tracing’, Schmid et al., 2025*)*. In the present work, we rather wished to train a network that is not hard-wired, to examine generalization to different tasks. The revision clarifies this point (p. 6-7, lines 131-137).

# R1-4) A similar disinhibitory circuit has been identified as an actor of contextual modulation (<https://www.cell.com/neuron/fulltext/S0896-6273(20)30891-6>). It can be interesting to comment on whether the current model aligns with or differs from observed activity modulation in context modulation.

# R1-4 reply) We thank the reviewer for this reference. We have updated the manuscript to discuss how our architecture aligns with the reported findings on the role of VIP neurons, SOM neurons and pyramidal neurons in the visual cortex during figure-ground segregation (p. 25-26, lines 539-544).

**Reviewer 2**

**General comment**

# R2-0) This is a strong and easy-to-read manuscript that provides a recurrent neural network implementation of a mechanistic account of the growth-cone model of perceptual grouping. The model performed well, reproducing findings from previous research, and I found particularly compelling its ability to generalize to unseen, more difficult task variants as well as to extend to a separate task, such as object parsing. I have only a few relatively minor comments and suggestions, mostly related to clarity and reproducibility.

# R2-0 reply) We are grateful to the reviewer for their thorough evaluation and positive assessment. Below, we provide a detailed point-by-point response to address the concerns.

# Comments

# R2-1)  I was unable to access the GitHub link to examine the data or code repository given in the “Data and Code Availability” statement. I recommend that the authors double-check the repository link and ensure all necessary materials are publicly available.

# R2-1 reply) We apologize for the inconvenience. The code repository is now public, alongside with documentation enabling others to replicate the training of these models.

# R2-2) It is explained that the neural network includes four spatial scales and qualitatively describes how receptive fields differ across them; however, the exact receptive field dimensions (e.g., kernel sizes, scaling relationship between layers, etc.) are not reported. Additionally, it would strengthen the biological interpretation to comment on whether the scaling of receptive field sizes across layers bears any relationship to RF scaling in the visual cortex.

# R2-2 reply) We have expanded the manuscript to provide additional architectural details, including a thorough description of connections within and between layers (p. 11-14, lines 238-313). We have also improved our description of the relationship between receptive field scaling across layers in our model and in the visual cortex (p. 13, lines 275-277).

R2-3) Figure 1, Panel B: The figure caption could be more descriptive. I am assuming the second panel is the difference between the target and distractor responses, but that is not explicitly stated in the figure caption.

R2-3 reply) We have improved the figure by including a schematic with what the stimulus looked like and updated the caption accordingly (p.8 lines 157-161)

R2-4) Figure 2, Panel B: The excitatory and inhibitory connections (circles vs. triangles) are difficult to differentiate at their size, especially in Layer l-1. Enlarging the symbols could improve readability.

R2-4 reply) We have updated Figure 2, enlarging the symbols.

**Reviewer 3**

**General comment**

R3-0) Mollard et al. propose a biologically plausible computational model of segmentation in the early visual cortex that performs well on (and generalizes between) two tasks: curve tracing, and naturalistic object parsing. The model uses a biologically motivated architecture in which recurrent interaction is implemented by disinhibition, rather than direct excitation, which allows the model to propagate activities over longer curves while keeping its activation stable. The model can reproduce several findings from psychophysics and neurophysiology, such as the slowing down of activity propagation in the presence of a nearby distractor curve. The paper is well written and motivated, and the results look solid and a meaningful improvement on the authors’ prior work (e.g. Mollard et al. 2024). There are, however, some issues which I believe the authors should address. In particular, I have a few major comments:

**R3-0 reply)** We thank the reviewer for his/her careful reading of our paper and their positive feedback. We review his/her comments point by point below.

**Major comments**

**R3-1)** Several details of the model architecture are unclear, particularly the feedforward component. In Eq. 1, it is clearly stated that the feedforward pass is based on convolutions. The details of these convolutional layers, however, such as the kernel size and stride and the number of channels, are not specified. Also, eq. 1 indicates the feedforward weights as FF_1, with no subscript or superscript corresponding to the layer. Does this mean that a single set of feedforward weights was shared across layers? I assume not, but if that is not the case the notation should be made clearer. I understand that this work is based on Mollard et al. 2024, and I imagine that many of the details are consistent with that paper (e.g. perhaps the 3x3 kernels enforcing a Von Neumann neighborhood), but there are several differences as well. For example, there was no separate training of the feedforward and feedback components in that previous paper. So I believe the authors should make it clearer in which aspects the two feedforward architectures differed and in which aspects they matched. In general, I think important details such as the exact architecture of the models are worth mentioning in this paper, even when they overlap with previous work, to make the paper more self-contained and easily readable. Finally, the authors should release the model’s code - there is a link, but it does not work (<https://github.com/samimol/multiscale_tracing>). Having access to the code would have disambiguated many of these architectural details.

# R3-1 reply) Thanks for bringing this up. We have now improved the description of the network architecture, detailing all the equations, including those used for network training. We expect that these changes make the manuscript self-contained (p. 9-14, lines 189-313). In addition, we have made the code repository publicly available, together with documentation enabling replication of all results, including the training procedures for the various models.

R3-2a) Related to the previous comment, also regarding the feedforward network, the way that the feedforward architecture’s task for its separate training was operationalized was not fully clear from the manuscript. My best guess as to how it worked is the following: an NxN grid (corresponding to the dimensions of the downsampled feature map after applying the convolutional layer, so dependent on the spatial scale) containing binary values (1 only if the contour within that location was fully colinear and connected, 0 otherwise) was provided to the network as a target. That was compared with the single layer’s output feature map using cross-entropy, and this error was backpropagated through the single convolutional layer. Is this how it was implemented? I assume that each convolutional layer’s output was a binary map, indicating whether a colinear edge was present in each unit’s RF. The authors should make this clearer, as it is not explicit in the text.

R3-2a reply) Yes, the reviewer’s interpretation of the feedforward architecture is correct. We have updated the manuscript accordingly, and provide more details (p.16-17, lines 340-359).

R3-2b) Here again, architectural details of the feedforward network do matter, as the supposed “locality” of the feedforward task (which the authors contrast with the spatial interactions happening in the recurrent part of the model) depends on the size of the convolutional kernels.

R3-2b reply) We now report the size of the convolutional kernels of the feedforward network (p.10, lines 203-217). We have also made it clearer that units in the feedforward network cannot solve the grouping tasks for stimuli extending beyond a limited neighborhood (p.17, lines 356-359).

R3-2c) Also, the fact that the different spatial scales were trained independently should be explained more explicitly, as this idea might be counterintuitive to readers (like myself) who might associate convolutional layers and backpropagation with a hierarchical convolutional architecture trained end-to-end, rather than each layer being trained separately on a different spatial scale.

R3-2c reply) We have now made the training of the various scales explicit (p.16-17, lines 340-359). We have also included a new figure panel Fig. 2D that illustrates this architecture.

R3-2d) Finally, was the separate training of the different spatial scales done with image inputs (i.e. subsampled versions of the same image, like those shown in figure 4D) or feeding each layer’s output to the layer above it? If each layer’s output was a binary map of edge presence, as I guessed above, then it would be possible to train the layer using image inputs and then deploy it on the outputs of the previous layers, but this is not clear from the manuscript.

R3-2d reply) The units at different scales were trained independently to detect homogeneous image regions (object parsing) or colinear contours (curve tracing task). There was a direct feedforward projection from the image inputs to each scale, so that the activity in e.g. layer 4 did not need to pass through layers 1, 2 and 3. We have now included a full description of the features of the feedforward networks (p.9-11 lines 188-219).

R3-3a) The model’s architecture is based on well-motivated biological principles, but it is not clear what the role of some its components is. One of these components is the two-stage training of the feedforward and recurrent networks. The authors mention that this choice was meant to be analogous to the tuning of feedforward neurons during visual experience. However, I believe they should clarify how crucial this separate training was to the results in the paper. As far as I can tell, the model in their 2024 paper was trained jointly using RELEARNN. Since the model in this paper builds upon that previous model (replacing excitation with disinhibition to generate more stable dynamics and allow longer-range enhancement), this mismatch complicates the comparison. Would the network still be able to effectively parse the input images if it were trained jointly? Or is it somehow the case that direct excitation allows the feedforward units’ selectivity to emerge through joint training, but this disinhibition mechanism doesn’t? The authors should clarify this point, if possible by attempting to train the whole system jointly. If that is not feasible, they should clarify why that is the case.

R3-3a reply) We thank the reviewer for this important point. We have attempted joint end-to-end training of the feedforward and recurrent networks using the same reinforcement learning procedure as in our previous single-scale model. Although disinhibitory propagation occurred in the higher layers, it was not reliably at the lower levels with the higher spatial resolutions. In other words, the network did not consistently develop scale-dependent grouping across all levels. In the multiscale setting, the training of feedforward and recurrent connections apparently imposes constraints that were not satisfied under joint training. We therefore adopted a two-stage training procedure, in which feedforward selectivity is established first and then held fixed during recurrent learning. We have clarified this point in the revised manuscript (p.26, lines 557–569).

R3-3b) Of course, the authors do include a more direct comparison (on p. 24, lines 486-488) with an excitatory network. This comparison is very informative, but I believe the authors should include more details about it, for example by visualizing both the ReLU and squashing models’ performance (e.g. heatmaps of their curve tracing, summary statistics of their performance) side by side with the disinhibitory network.

R3-3b reply) We thank the reviewer for this suggestion. We have now included a direct comparison between the new network and the previous purely excitatory recurrent networks from Mollard et al. (2024) as Supplementary Information. This new section includes side-by-side visualizations of performance and neural activity dynamics for the excitatory model and the present disinhibitory model.

Specifically, we now show (1) generalization performance for curves longer than those used during training, (2) example activity maps illustrating propagation along target and distractor curves, and (3) the temporal dynamics of the activity difference between target and distractor units as a function of distance from the fixation point. These analyses demonstrate that excitatory networks with squashing nonlinearities exhibit progressive attenuation of the modulation signal along the curve, impairing generalization to longer curves. In contrast, the disinhibitory architecture maintains a stable, non-attenuating signal and generalizes to longer curves.

R3-4) Another architectural component the role of which is not clear are the skip connections from each layer to the output layer. I believe the presence of skip connections complicates the interpretation of the feedback connections’ role in mediating interactions across scales. It is possible, for instance, that the need for cross-scale integration is reduced by the fact that the network can combine the outputs of different layers using skip connections. The authors should discuss what the functional role of the skip connections is, and their relation to other architectural components (especially feedback connections) if possible also trying to ablate them.

R3-4 reply) The skip connections do not eliminate the need for cross-scale integration. Units in layer $l$ project via skip connections to the $3^{l}\times3^{l}$ output units overlapping with its receptive field. The skip connections do not enable integration across adjacent receptive fields or distant image regions. We have clarified this point in the manuscript (p. 14, lines 309-313).

The skip connections ensure that the output layer has access to information from all scales. In their absence, the higher layers, which carry strong low resolution signals dominated eye movement selection. As a result, the network often did not select the correct pixel for an eye movement because the other pixels their large receptive fields had an equal selection probability. The skip connections preserved fine-grained spatial information at the output layer while still allowing coarser-scale grouping features to influence the decision. Hence, skip connections improve the precision of spatial readout, whereas feedback and horizontal connections remain essential for multiscale grouping and propagation of enhanced activity. We have now clarified the role of these connections (p. 14 lines 309-313).

**Minor comments**

 R3-5) A potentially relevant paper to cite in the introduction, where previous work on disinhibition in segmentation and attentional selection is mentioned (lines 206-207), is the following:

Linsley, D., Malik, G., Kim, J., Govindarajan, L. N., Mingolla, E., & Serre, T. (2021). Tracking without re-recognition in humans and machines. *Advances in neural information processing systems*, *34*, 19473-19486.

That paper incorporated a disinhibitory mechanism into a recurrent neural network trained to track objects across time in synthetic and natural videos. I see some analogy between the approach of this manuscript and that paper: where this manuscript describes a selective spread of activity across space, that paper does it across time. However, this is just a suggestion: this is not my field of expertise and there might be several more relevant papers which use similar mechanisms.

R3-5 reply) We thank the reviewer for his suggestion. We have incorporated this reference in the discussion (p. 26, lines 546-548)

R3-6) Beyond including more details on the feedforward architecture (which I have already mentioned above), the authors should include some more details on its training procedure, such as the number of iterations, and the criterion for stopping.

R3-6 reply) We have now also added more details about the training procedure of the feedforward network (p.16-17, lines 340-359)

R3-7) On p. 14, lines 281-282, “the activity of input neurons and units of the feedforward network determines the propagation of activity until convergence to a stable state”. I did not understand what this means exactly - isn’t the convergence determined by the activity of the full network, including the recurrent connections? The authors should clarify this.

R3-7 reply) Yes, thanks for pointing out that this formulation might cause confusion. The convergence to a stable state is indeed determined by the dynamics of the full network, including its recurrent connections. We have clarified that the recurrent network needs to converge in the revision (p. 17, lines 363-370).

R3-8) On p. 15, lines 313-314, “All networks converged (criterion of 85% correct) within an average of 23,200 trials”. Could the authors include more information (perhaps a supplementary plot) about the eventual accuracy which the networks reached, and how many iterations each of them took to reach it? Especially because the rest of the text seems to imply that the networks were 100% accurate in the task, so it would be useful to clarify this.

R3-8 reply) The networks were indeed 100% accurate in the task. They learnt a general solution to the curve tracing problem, which worked for every stimulus that we tried. We modified the text to clarify this point (p. 18, lines 398-399).

R3-9) On p. 16, lines 322-323: “Through trial-and-error learning, the network learns that the target curve begins with a red pixel. Specifically, learning increases the feedforward weights from the red pixel start so that the units that represent this location overcome SOM inhibition”. Perhaps this will become clearer when the authors clarify the details of the feedforward architecture that I mentioned previously, but how does this work precisely? Does it mean that the convolutional kernel has higher weights for one of the input channels (corresponding to the color red)?

R3-9 reply) The reviewer is correct because the weights between units representing the red color increased during learning. We modified the text accordingly (p. 19, lines 408-410).

R3-10) P. 18, line 375: in the text a noise ceiling of 67% is mentioned, but in Figure 4C, the red line is above 70%. Could the authors clarify this discrepancy?

R3-10 reply) We thank the reviewer for the careful observation. The noise ceiling was at 67% and we corrected the figure accordingly.

R3-11) Related to the comparison of the present model with the growth-cone model, shown in Figure 4C, do the authors expect that increasing the number of layers further would make the model reach the growth-cone model’s explained variance, or perhaps even surpass it, and reach the noise ceiling? Would it be possible to test this?

R3-11 reply) Increasing the number of layers would not necessarily improve the fit to the human data. Specifically, the GC model assumes a continuum of scales and cone sizes, whereas the RFs of the modeled network were discrete and increased in size across the layers by powers of three. Simply adding the next scale without modification of the kernels would further increase receptive field size (from 81x81 to 243x243 pixels) which would not improve the amount of explained variance. To approximate the performance of the GC model it might be necessary to modify the shape of the kernels and the logic of the interactions between layers representing different scales.
